# Social networks and mental health in post-conflict Mitrovica, Kosova

**DOI:** 10.1186/1471-2458-14-1169

**Published:** 2014-11-17

**Authors:** Risa Nakayama, Ai Koyanagi, Andrew Stickley, Tetsuo Kondo, Stuart Gilmour, Aliriza Arenliu, Kenji Shibuya

**Affiliations:** Department of Global Health Policy, Graduate School of Medicine, University of Tokyo, 7-3-1 Hongo, Bunkyo-ku, Tokyo, 113-0033 Japan; Department of Human Ecology, Graduate School of Medicine, University of Tokyo, 7-3-1 Hongo, Bunkyo-ku, Tokyo, 113-0033 Japan; United Nations Development Programme, Fada, Chad; Department of Psychology, University of Pristina, Pristina, 10000 Republic of Kosovo

## Abstract

**Background:**

To investigate the relation between social networks and mental health in the post-conflict municipality of Mitrovica, Kosovo.

**Methods:**

Using a three-stage stratified sampling method, 1239 respondents aged 16 years or above were recruited in the Greater Mitrovica region. Social network depth was measured by the frequency of contacts with friends, relatives and strangers. Depression and anxiety were measured using the Hospital Anxiety and Depression Scale (HADS). Multivariate logistic regression was used to examine the association between social network depth and mental health.

**Results:**

The analytical sample consisted of 993 respondents. The prevalence of depression (54.3%) and anxiety (64.4%) were extremely high. In multiple regression analysis, a lower depth of social network (contact with friends) was associated with higher levels of both depression and anxiety.

**Conclusions:**

This study has shown that only one variety of social network – contact with friends – was important in terms of mental health outcomes in a population living in an area heavily affected by conflict. This suggests that the relation between social networks and mental health may be complex in that the effects of different forms of social network on mental health are not uniform and may depend on the way social networks are operationalised and the particular context in which the relationship is examined.

## Background

During the past decade a growing body of research has suggested that there is a relation between a variety of health outcomes and social capital, which is understood as the resources that people can obtain through social networks [1, 2]. At both ecological and individual levels, social capital has been linked, after the adjustment for potential confounders, with mortality rates, life expectancy, suicide and homicide rates, sexually transmitted infections and self-rated health [3]. It has also been claimed that social capital may be important for mental wellbeing [4, 5]. At the individual level, social capital as measured by feelings of trust and social connections has been linked to better health through reductions in psychological distress [6]. This relation has been observed across different countries and age groups as two recent studies have also shown how low levels of social capital (interpersonal trust) are associated with higher levels of depression among both the young (Swedish adolescents) [7] and the elderly (Japanese) [8].

The finding that social capital is associated with mental health outcomes has not been universal, with a recent study showing no relation with major depression [9] while another study from four low income countries found that the effects of social capital on mental health outcomes varied depending on the specific form of social capital and the setting [10]. If the social capital-mental health relation does have setting-specific effects there are several factors, which may underpin this phenomenon, but differences in study design, confounder adjustment, and choice of ecological- compared to individual-level effects has made it difficult to separate the relative importance of setting or environment and individual factors in mediating the relationship between social capital and mental health.

The social environment not only influences factors that predispose individuals for mental illness, but also those which might be protective against its development [11, 12]. Moreover, previous research has shown that social capital is not homogenously available to everyone in a specific geographical location [13] but can vary in terms of its levels and forms between different individuals and groups [14] with the potential this might carry for differing effects in terms of mental health outcomes both within and between different settings.

The current study examined the association between one component of social capital, social networks, and mental health in Mitrovica, Kosovo. This location provides an ideal setting to examine this relation. A long history of inter-ethnic rivalry and tension between the Serbian and Albanian populations in Kosovo exploded into war in 1998-1999, resulting in over 10,000 deaths [15] with hundreds of thousands of people being internally displaced. Although the war was brought to an end in June 1999 following the intervention of the North Atlantic Treaty Organization (NATO) forces, its effects are still being felt today. Not only did the war result in a deterioration in population mental wellbeing [16] that has seemingly stretched beyond the war [17], but the continued dispute over the region’s ownership and accompanying periodic outbreaks of ethnic violence act to exacerbate a range of daily stressors (e.g. high levels of unemployment), that it has been argued can be important for social disruption and mental health in post-conflict settings [18, 19]. As yet, however, the effects of social networks remain little researched in Kosovo [20] either in terms of health or other outcomes.

In the current study, we use the work of people such as Nan Lin [1] as a base to conceive of social networks as the mechanism by which people can access/gain social capital within communities, with essential elements of social networks including trust in others (social cohesion), social connections, social support, and social integration [21, 22]. We also use the concept of bridging social networks, which measures contact with distant, little-known individuals.

Although it would have been desirable to analyze social capital at both the aggregate *and* individual levels, the current study focused on social networks at the individual level. It was not possible to use several measures that are normally aggregated in social capital studies as there are dangers associated with different forms of organizational gathering in this post-conflict setting [18, 19]. We found that organizational membership was extremely low. Moreover, other conventional practices were also difficult to quantify, as averaging measures of social capital among very different groups with very different social and behavioral practices (such as church attendance, voting practice etc) may have produced misleading results. Individual measures of social capital have been employed in several previous studies examining the relationship between the depth of social networks and mental health outcomes in different settings [23, 24]. In addition, in an environment where networks may have been destroyed due to conflict and the death and displacement associated with it [15, 25], links that stretch beyond immediate networks may also be important for mental health. Some previous research in other ‘distressing’ (non-conflict and conflict) settings supports this hypothesis [23, 24]. This focus on the importance and effects of social networks on mental health is operationalized in this study by exploring the effects of ‘informal social networks’ on mental health. Specifically, following the lead of a recent study by Ferlander and Mäkinen [26] this study examine how strong social ties such as the bonds between family and friends impact on mental health, and explore the relationship between bridging social networks and mental health.

## Methods

### Study site

The current study was undertaken in Mitrovica, Kosovo, from October to December, 2010. The municipality lies 40 kilometres north of the capital, Pristina. Since the war ended in 1999 both the municipality and the city of Mitrovica itself have been divided along ethnic lines [25] by the River Ibar. In 2009 there were an estimated 110,000 people in South Mitrovica, primarily Albanians but with minority populations of Bosniaks, Turks, Roma, Ashkali and Egyptians (RAE) and Gorani. North Mitrovica consists of 17,000 Serbians and 3000 other persons including Albanians, Turks, Gorani and RAE [27]. Since the 1999 ceasefire, the Nato-led Kosovo Force (KFOR) has maintained a strong presence in Kosovo, especially in Mitrovica city itself, guarding and monitoring the bridges linking the two halves of the city to prevent hostilities. However, tensions still run high as witnessed by the outbreak of violence which occurred in Mitrovica district in February 2008 following the declaration of independence by Kosovo’s Albanian population [25].

### Sampling methodology

The data used in the present study come from a survey conducted by the United Nations Development Programme (UNDP) Kosovo in October 2010 [27]. Using a three-stage stratified representative sampling technique, 1239 respondents aged 16 and above were recruited into the study within polling station territory in North and South Mitrovica. With settlement type (urban/rural) as the unit of stratification, polling station territories where more than 10% of all registered voters voted in 2009 were designated as the primary sampling units (PSUs) and selected with probability proportional to size (PPS). Within PSUs the secondary sampling units (SSUs) were households which were selected using a random walk technique. The random walk technique was conducted by providing the interviewers with the following instructions: from a given starting street and house number, the interviewer headed in the instructed direction aiming to identify the first dwelling on their path. When they entered the selected dwelling, they followed a standard procedure for choosing one apartment randomly. The tertiary sampling unit were the respondents within households, selected with the use of Kish tables. One respondent was chosen from each household. Face-to-face interviews were conducted by trained interviewers using a questionnaire containing sections on demographic characteristics, aspects of daily life, use of media, health behaviours and physical and mental wellbeing. The questionnaire was developed using standard back translation techniques with any discrepancies being resolved following discussion among experts. Verbal and written informed consent was obtained from all participants. The final response rate was 80.1%.

### Ethical approval

Ethical approval for the study was obtained from the Ethics Committee at the University of Tokyo. UNDP Kosovo also approved the protocol.

### Covariates

Following on from earlier research on health outcomes among conflict-affected populations [28], information was collected on the age, sex and ethnicity of respondents, on their marital status (single, married, divorced/separated), highest educational level (primary school, secondary school or university), place of residence (North or South), and level of household wealth. Principal component analysis (PCA) was used to generate a household wealth index based on the ownership of items from a list of household assets. As the study population was relatively small, the wealth variable was divided into tertiles (poor, middle, rich) rather than quintiles.

The current study followed the lead of a recent study examining social capital and self-rated health in Moscow, which focused on the role of informal social networks (i.e. contacts with family, friends and relatives) [26, 29]. In addition, information was also obtained on a ‘bridging’ form of social relation - contact with other people little known to the respondents. We asked the interviewees how frequently they had contact with strangers, friends, and relatives with the following questions: 1) “How often do you meet with people that you do not know particularly well?” 2) “How often do you meet a friend as a leisure activity?”, 3) “How often do you meet your relatives as a leisure activity?” where the answer options were often, sometimes or never. This focus on the structural (associational) forms of social networks was driven by earlier research which has suggested that familial networks might be potentially important components for mental health outcomes in Kosovo [30].

The Hospital Anxiety and Depression Scale (HADS) questionnaire was used to identify individuals with depression and anxiety. The scale consists of 14 questions with scores ranging from 0 to 3 for each question, with two subscales for anxiety and depression. Scores were summed for each subscale and a cut-off point of ≥8 was used to identify cases of anxiety and depression [31]. This instrument was previously used in a UNICEF survey in Kosovo in 2009 [32], with Cronbach α scores ranging from 0.50 (for anxiety in Albanians) to 0.76 (depression in Serbians). In this study, these instruments were self-reported measures and therefore, we revalidated the instrument before it was used in our study as the low Cronbach’s alpha obtained for the Albanian version of the anxiety subscale raised concerns. A revision of the anxiety subscale was undertaken which identified that item 11 (with the response option “I am continuously moving and I can't stand in one place”) was the major contributor to the low Cronbach’s alpha obtained. After local Albanian psychologists retranslated the scale, it was pilot tested with 43 students at Prishtina University to examine its reliability. Through retranslation of item 11, the Cronbach’s α of the HADS scale in Albanian increased considerably from 0.50 to 0.68 for the anxiety subscale and 0.65 to 0.72 for the depression subscale.

### Statistical analysis

Statistical analyses were conducted using Stata version 11.1 (StataCorp LP, College Station, TX). Multivariate logistic regression models were constructed to examine the association between social network depth and depression and anxiety (scored 0 = no 1 = yes). In the analysis, for the three social network variables, the response categories “sometimes” and “never” were combined since their frequencies were too low to allow them to be analyzed as separate categories. The analyses were undertaken using a step-wise model where the social network variables and those for sex and age were retained in all models. All regression analyses were adjusted for the effect of the probability sampling structure of the survey. The results are presented in the form of odds ratios (OR) with 95% confidence intervals (CI). The level of statistical significance was set at p < 0.05.

## Results

A total of 1239 individuals were recruited into the study, but 244 withdrew or failed to answer essential questions, leaving 993 individuals for analysis (response rate 80.1%). Baseline characteristics of the study population are presented in Table 1. Around 75% of the respondents were aged under 50 with over 60% of the sample being married. The majority of both men and women had received a secondary education. The prevalence of psychological illness was exceptionally high among both sexes. Over 50% of the population (54.3%) was categorized as having depression, while the figures were even higher for anxiety with 64.4%.

**Table 1:** **Baseline characteristics of the study population**

Variable	Category	N	%	(95% CI)
Sex	Male	498	50.2	(44.8 - 55.5)
	Female	495	49.9	(44.5 - 55.2)
Age	<30	356	35.9	(32.1 - 39.6)
	30-49	393	39.6	(33.6 - 45.5)
	> = 50	244	24.6	(20.9 - 28.2)
Education	University	96	9.8	(6.9 - 12.7)
	Secondary	593	60.5	(53.0 - 67.9)
	Primary	292	29.8	(21.9 - 37.6)
Marital	Married	617	62.9	(59.2 - 66.6)
	Single	308	31.4	(27.8 - 35.0)
	Divorced	56	5.7	(3.9 - 7.5)
Wealth	Rich	323	32.7	(28.3 - 39.6)
	Middle	335	33.9	(25.1 - 41.6)
	Poor	329	33.3	(27.5 - 38.0)
Ethnicity	Albanian	497	50.1	(29.5 - 70.6)
	Serbian	300	30.2	(6.6 - 53.9)
	Minority	196	19.7	(5.5 - 34.0)
Municipality	North	484	48.7	(26.4 - 71.1)
	South	509	51.3	(28.9 - 73.6)
Depression	No	448	45.7	(38.0 - 53.4)
	Yes	532	54.3	(46.6 - 62.0)
Anxiety	No	350	35.6	(29.4 - 41.8)
	Yes	633	64.4	(58.2 - 70.6)

Total number of respondents 993.

Table 2 presents details of subjects’ social network scores. The number of individuals who answered “never” for the ‘contact with friends’ (n = 42) and ‘relatives’ (n = 16) questions was quite small. As a result of this we subsequently combined the categories “sometimes” and “never” in the logistic regression analysis. For ‘contact with relatives’ and ‘friends’, most of the respondents answered with the category “often” (70.8% for relatives, 53.2% for friends). In contrast, in terms of ‘contact with strangers’ the response category which predominated was ‘sometimes’ (64.0%) with very few of the respondents making contact with strangers ‘often’ (18.3%).

**Table 2:** **Social networks**

Social network type	Often	%	Sometimes	%	Never	%
Contact with relatives	703	70.8	274	27.6	16	1.6
Contact with friends	528	53.2	423	42.6	42	4.2
Contact with strangers	182	18.3	635	64.0	176	17.7

Total number of respondents 993.

The prevalence of the two mental health outcomes by social network depth are presented in Table 3. For both depression and anxiety there were significant differences in terms of the prevalence of depression and anxiety for two of the three social network categories. Meeting with friends and strangers less often was associated with a significantly higher prevalence of depression. Similarly, the prevalence of anxiety was significantly higher for people who met with relatives and friends less frequently.

**Table 3:** **Social networks and anxiety and depression**

		Depression					Anxiety				
Social network type		No	%	Yes	%	p-value	No	%	Yes	%	p-value
Contact with relatives	Often	311	70.2	381	73.1	0.5	268	77.7	428	68.7	0.04
	Sometimes/never	132	29.8	140	26.9		77	22.3	195	31.3	
Contact with friends	Often	276	62.6	246	49.5	0.01	243	71.9	281	46.6	0.00
	Sometimes/never	165	37.4	251	50.5		95	28.1	322	53.4	
Contact with strangers	Often	98	21.9	82	15.4	0.05	69	19.7	113	17.9	0.5
	Never/sometimes	350	78.1	450	84.6		281	80.3	520	82.2	

Note: Anxiety was defined as score ≥ 8 on the HADS scale. Total number of respondents 993.

The results of the multiple regression analysis of the association between social network depth and mental health outcomes are presented in Tables 4 and 5. For depression, those individuals who made contact with friends ‘sometimes/never’ had a 2.41 higher odds (95% CI: 1.47-3.94) of experiencing depression compared with those who met friends ‘often’. Wealth, municipality, and marital status were also associated with depression: respondents who were poor had a 2.82 times (95% CI: 1.52-5.22) higher risk of depression compared to those who were rich; living in the South was associated with a 37% reduction in the odds of experiencing depression (OR: 0.63; 95% CI: 0.41-0.96); while being single or divorced was associated with a 53% (OR: 0.47; 95% CI: 0.28-0.78) and 55% (OR: 0.45; 95% CI: 0.23-0.86) reduction in the odds of having depression respectively.

**Table 4:** **Multivariate logistic regression of the association between social networks and depression**

		Depression	
Variable	Category	OR (95% CI)	P-value
Contact with relatives	Often	1.00	
	Sometimes/never	0.61 (0.36-1.03)	0.07
Contact with friends	Often	1.00	
	Sometimes/never	2.41 (1.47-3.94)	<0.001
Contact with strangers	Often	1.00	
	Sometimes/never	1.33 (0.86-2.05)	0.2
Sex	Male	1.00	
	Female	0.87 (0.57-1.33)	0.5
Age	>30	1.00	
	30-40	0.70 (0.39-1.27)	0.2
	> = 50	0.77 (0.33-1.84)	0.6
Marital status	Married	1.00	
	Single	0.47 (0.28-0.78)	0.01
	Divorced	0.45 (0.23-0.86)	0.02
Wealth	Rich	1.00	
	Middle	0.97 (0.58-1.62)	0.9
	Poor	2.82 (1.52-5.22)	<0.001
Municipality	North	1.00	
	South	0.63 (0.41-0.96)	0.03

Note: Depression was defined as score ≥8 on the HADS scale. Total number of respondents 993.

**Table 5:** **Multivariate logistic regression of the association between social networks and anxiety**

		Anxiety	
Variable	Category	OR (95% CI)	P-value
Contact with relatives	Often	1	
	Sometimes/never	1.02 (0.63-1.65)	0.9
Contact with friends	Often	1	
	Sometimes/never	2.43 (1.61-3.66)	<0.001
Contact with strangers	Often	1	
	Sometimes/never	1.00 (0.67-1.48)	1.0
Sex	Male	1	
	Female	1.06 (0.72-1.55)	0.8
Age	>30	1	
	30-40	0.67 (0.39-1.14)	0.1
	> = 50	0.63 (0.36-1.12)	0.1
Marital status	Married	1	
	Single	0.47 (0.28-0.78)	0.01
	Divorced	0.90 (0.43-1.91)	0.8
Ethnicity	Albanian	1	
	Serbian	0.50 (0.34-0.74)	<0.001
	Minority	0.72 (0.43-1.22)	0.2

Note: Anxiety was defined as score ≥8 on the HADS scale. Total number of respondents 993.

For anxiety, in terms of social networks, the same results were observed as with depression – only contact with friends was associated with anxiety, with those individuals who met with friends ‘sometimes/never’ having 2.43 times the risk of experiencing depression compared with those individuals who met friends often. Being single was associated with a 53% reduction in the odds of experiencing anxiety (OR: 0.47; CI: 0.28-0.78), while Serbians had a significantly reduced risk of experiencing anxiety when compared to Albanians (OR: 0.50; CI: 0.34-0.74).

## Discussion

In the presence of extremely high levels of mental ill health in Kosovo, a number of demographic and socioeconomic factors were associated with having poorer mental health. Of the three social network variables examined in this study, only one of them – contact with friends – was associated with mental health, with those respondents who met with friends less often having a significantly higher risk of experiencing both depression and anxiety.

Although previous research indicates that the prevalence of mental illness may be higher in Kosovo than in other war-affected Balkan countries [33] the finding that 55% to 65% of the respondents suffered from poor mental health is high compared to earlier studies in Kosovo [34, 35]. Previous research has, however, highlighted that different forms of mental illness vary significantly by location in Kosovo [35], and previous surveys were conducted in different regions, using different instruments to assess mental illness, so may not be comparable. These surveys were also conducted before the increase in ethnic violence observed in 2009. In this respect, the current situation in Mitrovica may be extremely detrimental to mental health. An upsurge in ethnic conflict since 2009 [35] may be exacerbating a number of everyday stressors that can affect mental health such as poverty and limited/no access to medical care [35]. Indeed, poverty is widespread in Mitrovica [25] and as our analysis showed is strongly connected with poorer mental health (depression) in this setting. Moreover, another phenomenon that might underpin the poverty-mental disorders relation – unemployment [33] – is also endemic in Mitrovica: a recent study reported that over 40% of all household heads were unemployed or performing unpaid work there [25].

In the present study we found that from the three forms of social networks examined, only contact with friends was associated with mental health. Having said this, for both outcomes, being married was associated with a greatly increased risk of poor mental health compared with those who are single (while the same applied to the married-divorced relation in terms of depression). This is important as a recent study has conceptualised marital status itself as a form of social capital [25]. Given this, a strong finding of this study is that informal family-based association has either no effect or might even be detrimental for mental health in Kosovo. Previous research has shown how ‘bonding’ social networks – ties to others who are similar – can actually be a source of mental distress [23] and that such ties may be especially damaging in conditions of poverty – where obligations and commitments can often be a source of severe stress [29]. It can be hypothesised that if the close or extended family is the site where the effects of war and a wide range of everyday stressors are dealt with then it might itself be an especially stressful environment. This is important as previous research has suggested that there is a close association between stress and poorer mental health [36].

Indeed, one source of this stress might arise from the fact that it is the family which is often the site where mental health problems are handled in Kosovo due to the shame and stigma associated with poor mental health in this setting [30], with the consequences of this for mental health possibly having been exacerbated in recent years by the effects of war acting to both increase mental health problems and limit other outside treatment options [35, 37]. If the supposition that the family environment is a source of potential stress more than support, and a precursor of poorer mental health is correct, it might also explain another of our findings – the fact that Albanians have significantly higher levels of anxiety than Serbians – as recent research has highlighted that Albanians have much larger families in Mitrovica [25] with the potential this might carry to generate increased levels of stress and thus poorer mental health.

In contrast, contact with friends might allow time to be spent away from the family environment– which might explain why having less contact with friends was itself associated with poorer mental health in the current study. This finding mirrors that of a recent study from Moscow where little contact with friends was associated with worse self-rated health [26]. There are several ways in which friendship networks might be associated with better mental health in Mitrovica. Contact with friends might be a source of entertainment and relaxation which can act to counter other sources of stress [26]. Friendship networks can also act as a source of support when it comes to dealing with stressors with previous research highlighting how having a close confidant lessens the chance of experiencing poor mental health when faced with traumatic life events [29]. It has also been suggested more generally, that social networks can provide other forms of support such as health-related informational support or help when it comes to obtaining informal health care [38]. All together this suggests that in an environment which has many socially and economically challenging aspects (ongoing ethnic tension, restricted mobility, high levels of unemployment and widespread poverty) and where an inability to access health care has been associated with poorer mental health [35], extra-familial friendship networks might thus be especially important resource which can protect against mental health problems.

Our results also indicate that there may be a ‘dark side’ to strong social networks, however, as those Albanians who met with relatives more often were significantly more likely to experience depression. This finding mirrors that from an earlier study in Croatia which showed that individuals who visited friends and relatives often and who had experienced property damage in the Croatian civil war were *more* likely to report war-related distress [39]. The notion that social networks and social relations may be harmful for health has been highlighted especially in relation to poverty where the importance of the informal social network increases due to such things as limited access to health care [29]. In such circumstances it has been noted that social relations have the potential to become oppressive [40]. As yet it is uncertain why this result was noted only for Albanian respondents. It is possible that it could be related to the more traditional nature of Albanian family life and family structure, which might be particularly burdensome for women for example. In particular, earlier research in other contexts has indicated that women in large networks are “*often involved in the stress of others, and thus experience more stress themselves than women with smaller networks or than men do*” [29], with the obvious risk that this carries for affecting their own mental health.

Finally, in all ethnic groups those people who met with strangers more often were more likely to have better mental health. In this study we have interpreted meeting with strangers as a form of ‘bridging’ social network i.e. “*relations of respect and mutuality between people who know they are not alike in some socio-demographic (or social identity) sense (differing by age, ethnic group, class etc)*” [41]. It has been suggested that such bridging ties might be useful for accessing new and different forms of information, leading to improvement in individuals’ problem-solving ability [29]. It can be hypothesized that this might impact on mental health in Mitrovica both directly and indirectly. For example, given the link between unemployment and mental health [42] access to information about employment opportunities and the work they might bring in an environment with high levels of unemployment might be associated with better mental health. It is also possible that these links may impact on mental health more directly by facilitating access to necessary medical services or by providing health information. Future research should examine the nature of these ‘bridging’ links more closely to determine how they are associated with better mental health.

Before concluding it is necessary to mention several possible study limitations. Our measure of social networks in this study is limited, does not address the full range of measures of social network strength and does not include measures of other forms of social connection, so should not be interpreted as equivalent to a full measure of social capital. This limitation partly derives from the post-conflict setting, in which it is difficult to collect information on aspects of organizational behaviour necessary to establish a full understanding of social capital; and partly due to the difficulty of defining social capital in areas where normal social relations have been disrupted by conflict. Further research on the best measures for defining social capital in conflict zones, and for collecting organizational behaviour information in a safe way, may be necessary to solve these problems in future. The cross-sectional nature of this study means that the direction of causality cannot be determined. For example, depression and anxiety may have been a cause of lower levels of contact with others. Indeed, a recent study has highlighted how poor mental health within families is sometimes associated with greater social isolation in Kosovo [30]. It was not possible to control for this problem by, for example the inclusion of instrumental variables because of the absence of suitable candidates for instrumental variable analysis in the small questionnaire. However, although the potential for a bidirectional relation exists, among the 12 longitudinal studies on the effect of social capital on mental health that we identified, nine studies demonstrated that social networks are protective against mental ill health [24, 43–50] with only three studies showing mixed results [45, 51, 52]. Residual confounding is also a potential problem in this study, as we could not adjust for variables such as war-related experiences, previous clinical history, alcohol or other drug use and unemployment which are all risk factors for mental disorders. Thus, the independent and confounding effects of these factors remain unknown. Another limitation in this study is the quality of the questionnaire, which was not translated into the minorities’ native languages and thus, various degrees of bias may have been introduced due to the respondent’s ability to understand Albanian or Serbian, which were the languages in which the survey was administered. However, this bias would likely only occur amongst a minority of (non-Albanian, non-Serbian) respondents, and interviewers were able to help them where necessary to reduce the effect of this problem. This questionnaire was also initially found to have some translation problems, and the validation process was only conducted on a small pilot group of 43 students, so it is possible that the instrument used to assess mental illness and social network strength in this study may not be equally valid across all types of study respondent, introducing bias in responses. Various degrees of bias may have therefore been introduced into the study.

## Conclusion

This study has shown that social network depth is associated with mental health in Mitrovica, Kosova. However, we also found the effects of social networks were not uniform. Specifically, greater contact with friends was associated with better mental health while contact with relatives and strangers had no effect on mental health. This result suggests that the relation between social networks and mental health is complex and depends on both the way social networks are operationalised and the setting in which the relation is examined. Psychologists and health workers operating in the long-term aftermath of conflict should be aware of the role social isolation may play in exacerbating mental illness.
